# Steps toward improving diet and exercise for cancer survivors (STRIDE): a quasi-randomised controlled trial protocol

**DOI:** 10.1186/1471-2407-14-428

**Published:** 2014-06-13

**Authors:** Lauren J Frensham, Dorota M Zarnowiecki, Gaynor Parfitt, Rebecca M Stanley, James Dollman

**Affiliations:** 1 Exercise for Health and Human Performance, School of Health Sciences, University of South Australia, Adelaide, SA 5000, Australia

**Keywords:** Physical activity, Walking, Pedometer, Cancer survivorship, Online intervention, Lifestyle intervention

## Abstract

**Background:**

Cancer survivorship rates have increased in developed countries largely due to population ageing and improvements in cancer care. Survivorship is a neglected phase of cancer treatment and is often associated with adverse physical and psychological effects. There is a need for broadly accessible, non-pharmacological measures that may prolong disease-free survival, reduce or alleviate co-morbidities and enhance quality of life. The aim of the Steps TowaRd Improving Diet and Exercise (STRIDE) study is to evaluate the effectiveness of an online-delivered physical activity intervention for increasing walking in cancer survivors living in metropolitan and rural areas of South Australia.

**Methods/Design:**

This is a quasi-randomised controlled trial. The intervention period is 12-weeks with 3-month follow-up. The trial will be conducted at a university setting and community health services in South Australia. Participants will be insufficiently active and aged 18 years or older. Participants will be randomly assigned to either the intervention or control group. All participants will receive a pedometer but only the intervention group will have access to the STRIDE website where they will report steps, affect and ratings of perceived exertion (RPE) during exercise daily. Researchers will use these variables to individualise weekly step goals to increase walking.

The primary outcome measure is steps per day. The secondary outcomes are a) health measures (anthropometric and physiological), b) dietary habits (consumption of core foods and non-core foods) and c) quality of life (QOL) including physical, psychological and social wellbeing. Measures will be collected at baseline, post-intervention and 3-month follow-up.

**Discussion:**

This protocol describes the implementation of a trial using an online resource to assist cancer survivors to become more physically active. It is an innovative tool that uses ratings of perceived exertion and daily affect to create individualised step goals for cancer survivors. The research findings may be of relevance to public health policy makers as an efficacious and inexpensive online-delivered intervention can have widespread application and may improve physical and psychological outcomes among this vulnerable population. Findings may indicate directions for the implementation of future physical activity interventions with this population.

**Trial registration:**

Australian New Zealand Clinical Trials Registry: ACTRN12613000473763.

## Background

The number of cancer survivors in Australia is increasing due to population ageing, growing incidence of some cancers and improvements in early detection and treatment [1]. However, cancer survivorship care is a neglected phase of the cancer care trajectory. The medical system tends to focus primarily on curing the cancer rather than managing the after-effects of treatment. Cancer and its treatments are often associated with adverse physical and psychological effects that can persist for months or years after treatment. Such after-effects can include fatigue, functional impairment, weight gain, sleeping difficulties and reduction in quality of life [2-7].

Increasing physical activity provides a non-pharmacologic strategy for the prevention and/or alleviation of many of these effects. Evidence has consistently indicated that physical activity has a positive impact on physiological outcomes (e.g. cardiovascular fitness, physical functioning, immune function, muscle strength, body composition, nausea and fatigue) and psychological wellbeing (e.g. mood, self-esteem, anxiety and depression) following a cancer diagnosis [8-13].

Despite these health benefits, many cancer survivors are not sufficiently physically active [14]. Cancer survivors living in rural and remote areas are at even higher risk of being physically inactive and experiencing negative effects after treatment than their urban counterparts [15,16]. This may be due to unique features of the rural environment including geographic diversity, social isolation, limited access to treatment services and facilities and the burden of travel distances [17]. Thus, there is a need for tailored, broadly accessible health promotion interventions to improve outcomes for survivors living in both metropolitan *and* rural areas.

The purpose of this paper is to describe the protocol of the STRIDE 12- week intervention, which has been specifically designed to test the effectiveness of an online resource for increasing regular walking and improving health and quality of life outcomes among cancer survivors living in metropolitan and rural areas. The trial also aims to identify whether there are region specific (metropolitan versus rural) predictors of successful engagement with the intervention. To our knowledge, it will be the first to use ratings of perceived exertion (RPE) and daily affect to deliver individualised, incremental step goals for participants relative to their baseline values.

### Theoretical framework

STRIDE is designed upon social cognitive theory which posits that physical activity interventions are most effective when they include intrapersonal mediators (including goal setting, self-monitoring and self-efficacy), social mediators (family and peer support), and environmental mediators (access to facilities and opportunities) [18-20]. Concepts from Locke and Latham’s [21] goal setting theory will also be integrated by encouraging 1) *goal acceptance* (participants will be provided with a workshop of the importance of active lifestyles thus placing importance on the achievement of their step goals); 2) *goal specificity* (step goals will be tailored to the individual taking into consideration their affect and level of physical capability); 3) *providing difficult goals* (step goals will be set high enough to encourage high performance but low enough to be attainable); and 4) *feedback* (a graph on the STRIDE website will show weekly step count averages over the 12 week program).

## Methods/Design

### Participants and setting

The STRIDE study is a quasi-randomised controlled intervention trial. Participants will be allocated to either the intervention group or the wait-list control group. Approximately 80 participants will be recruited and randomised by the research investigator(s). Table 1 shows the inclusion and exclusion criteria.

**Table 1:** Inclusion and exclusion criteria

**Inclusion criteria**	**Exclusion criteria**
• Age over 18 years, no age limit higher	• Patient with metastasis,
• Have had cancer treated with curative intent (excluding skin cancer)	• Pregnant or intending to become pregnant during study period,
• Not undergoing active treatment (including chemotherapy and radiotherapy but will be included if taking long-term follow-up medications or therapies such as hormone therapy)	• Physical or psychological condition (i.e. cognitive decline) that may impede their participant in the study
• Permanent resident of South Australia,	
• Insufficiently active (engaging in less than 20 sessions of physical activity, one session lasting 30 minutes) over the past month determined by The Active Australia Survey [22]	
• Regular access to the internet (whether it be personal access or through a library or community centre)	
• Sufficient English language skills and cognitive ability to complete questionnaires,	
• Satisfy stage one of the pre-exercise screening guidelines for commencing exercise determined by the Sports Medicine Australia Pre-Exercise Screening System [23]	
• Approval by treating doctor to be part of the study,	
• Provide written informed consent	

### Study design and recruitment procedures

#### *Recruitment and screening*

**Figure 1:**
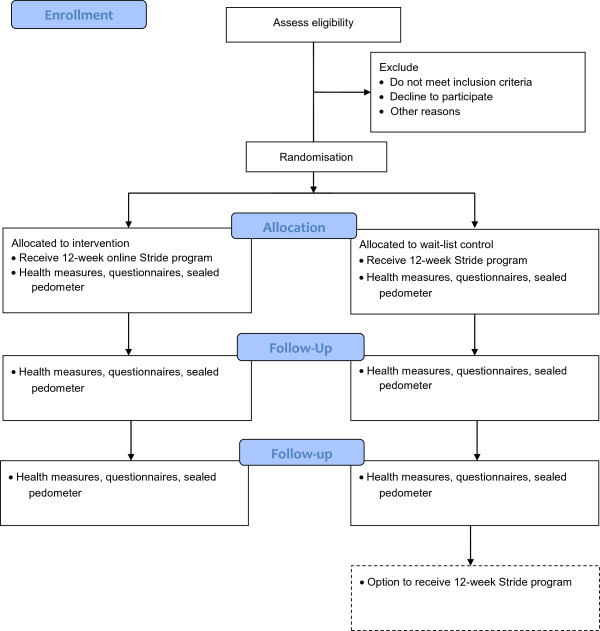
Flow of participants in study.

The study flow is presented in Figure 1. Participants will be recruited via cancer support groups, newspaper advertisements, hospitals, flyers, government departments and allied health personnel contacts. Potential participants will firstly be screened by telephone to determine eligibility, and if eligible will be provided with further study information, a consent form and pre-exercise screening form, and asked to obtain medical clearance from their treating doctor using a standardised medical clearance form. Pre-exercise screening will be conducted using Stage 1 of the Sports Medicine Australia Pre-Exercise Screening System [23]. The first stage of the screening system is designed to screen out those people who are at a high risk for exercise-related complications due to underlying cardiovascular, cerebrovascular, respiratory or metabolic diseases and also pre-existing injuries. Upon completion and receipt of required consent and clearance forms by the research team, participants will be randomised into treatment conditions and contacted to make arrangements for study participation.

### Randomisation and blinding

Participants will be randomly allocated to one of the two trial arms (intervention or wait-list control). A block design with allocation weight of 3:3 will be used to generate treatment allocation. It is not possible for three key researchers (LF, DZ, RS) to be blinded to participants’ group allocation as they will be performing the information sessions relevant to each group. It is not possible to blind participants to group allocation due to the nature of the intervention. Six other research assistants performing physical and questionnaire measurements will be blinded to the participants’ group allocation.

### Ethical approval

This study has received ethical approval from the University of South Australia Human Research Ethics Committee prior to study commencement and is registered with the Australian New Zealand Clinical Trials Registry (Trial Registration: ACTRN12613000473763).

### Protocol

All participants will attend two baseline workshops, one week apart. Each session will last approximately 90 minutes. At the first workshop, health measures will be taken (blood pressure, weight, height, waist and hip circumference) and fitness will be assessed using the 6-minute walk test. Participants will complete questionnaires about their physical functioning, quality of life and diet habits. They will be provided with a sealed pedometer (New-Lifestyles NL-1000 pedometer, New Lifestyles Inc., Lees Summit, MO) to wear for seven consecutive days (5 week days and 2 weekend days) which they will return at session two. At session two all participants will be provided with lifestyle information and a pedometer for use in the intervention (Yamax Digiwalker SW700). Those in the intervention group will be instructed on using the STRIDE website including how to log their steps and report their RPE and affect each day. On the basis of this information, they will be emailed weekly step goals which they will be encouraged to achieve. Following the 12-weeks of the intervention period, all measures will be repeated, and again after 6-months (i.e. 3-months post-intervention). At the conclusion of 6-months the control group will be offered the STRIDE program.

### Intervention – STRIDE website

The STRIDE intervention involves two key components, the STRIDE website and weekly step goals which participants are encouraged to achieve daily. Participants use a pedometer to monitor the number of steps taken each day, and record their daily steps using the step log on the STRIDE website. In addition to steps taken, participants will report their RPE and affect daily (described below). The step log screen will include a graph of average weekly steps which provides participants with feedback on progress throughout the intervention. There will also be an online forum through which participants can share experiences and provide peer support. Social support has been consistently reported as a predictor of maintained behaviour change in lifestyle promotion [24-28]. A virtual notice board will provide space for community service providers such as community centres, health services and walking groups to advertise events and activities, as well as provide high quality evidence-based guidance on lifestyle behaviours. This feature will be designed to reduce environmental barriers by increasing access to safe, supervised and socially focused opportunities for cancer survivors and family members. The website will also include information on healthy eating based on the Cancer Council Australia’s nutrition guidelines that in turn are based on recommendations in The Australian Guide to Healthy Eating [29].

### Step goals

Personalised step targets will be created using individuals’ RPE and affect. RPE is determined using Borg’s (1998) 6–20 Ratings of Perceived Exertion (RPE) scale [30] that numerically quantifies the effort, strain, discomfort and/or fatigue experienced during physical activity to represent how difficult or easy the activity is perceived to be [30]. RPE has been used to create target exercise intensities in a range of populations including healthy adults [31,32], cardiac patients [33] and patients with chronic obstructive pulmonary disease [34-36]. Researchers have suggested that RPE can be beneficial to elderly or patient populations where direct methods using maximal exertion are considered unsafe [37]. Research has indicated that affective state (feeling good/bad) influences exercise behaviour and motivation [38]. Given that a population such as cancer survivors may experience wide variability in affective state, how the individual is feeling will be taken into account when setting the individualised step goals. Participants will rate their affective state (i.e. how they feel [good or bad]) each day on a scale of +5 (‘Very good’) to -5 (‘Very bad’) [39].

Researchers on the STRIDE research team will use these inputs (daily steps, RPE during walking and daily affect) to generate individually tailored target steps/day for the following week that maintain exertion at between RPE 11 (light) and 13 (somewhat hard) on the RPE scale, the “bandwidth” within which people have the most positive response to exercise [40-42]. Three-tiered step goals will be provided to participants using the affect scale – a goal for when the participant is feeling ‘bad’ (i.e. minus on the affect scale), ‘neutral’ and ‘good’. This method will be employed to ensure that the goals are perceived to be challenging yet achievable, as recommended by Locke and Latham [21], even with variability in affective state from one day to the next. That is, the goals will take into account how the individual feels and be higher when affect is ‘good’ than ‘neutral’ and ‘bad’.

The step goals will be determined from steps, RPE and affect reported in the previous week. If participants do not meet the step goals set for the previous week on most days, the goal for the following week will not change from the previous week. The first week will serve as baseline data on which to set the first goals, with 0%, 5% and 10% increments on average steps achieved set for ‘bad’, neutral’ and ‘good’ days respectively. Weekly goals were set by LF and DZ and confirmed by JD and GP.

### Outcome measures

Outcome measures will be assessed at three time points: baseline, end-program (week 13) and at the 3-month follow-up. The primary outcome is *daily steps*. Participants will wear a sealed pedometer (New-Lifestyles NL-1000 pedometer, New Lifestyles Inc., Lees Summit, MO) for seven consecutive days (5 week days and 2 weekend days), except for sleeping, showering, bathing, swimming or engaging in contact sports. Minimum wear time, as recorded by a log sheet, will be defined as 10 hours per day for four of the seven days, one of which must be a weekend day. The NL series pedometer has extensive empirical backing in the literature for validity and reliability, and displays the accuracy required to detect changes in step counts that are typical of walking interventions [43].

Secondary outcomes include:

#### *Anthropometry*

The following anthropometric measures will be taken: standing stretch stature using a portable stadiometer (SECA, Hamburg, Germany); body weight (Tanita UM-108, Tanita Corporation, China); and waist and hip girths (Executive Thinline 2 m W606pm, Lufkin Tape, Apex, NC). All physical measurements will be taken according to the International Standards for Anthropometric Assessment [44]. A minimum of two measures will be taken at each site. A third measure will be taken if the difference between the first and second measures is greater than 0.5 cm for stature, waist and hip girths.

#### *Physiological measures*

Resting blood pressure will be measured after participants have been seated for five minutes using automated sphygmomanometers (Dinamap Pro 100, GE Medical Systems Information Technologies and Critkon Company, LLC, United Kingdom). Measures will be repeated until there are two readings within 5 mmHg for both systolic and diastolic pressure, up to a maximum of four times.

#### *Diet quality*

A semi-quantitative Dietary Questionnaire for Epidemiological Studies (DQES) developed by the Cancer Council of Victoria [29] will be used to assess change in dietary habits. The DQES asks about food intake over the previous 12 months. It contains 74 items with 10 frequency response options ranging from “Never” to “3 or more times per day”. The questionnaire also contains three photographs of scaled portions of four foods, questions on the overall frequency of fruit and vegetable consumption and questions on consumption of foods that do not fit easily into the frequency format, such as breads.

The 74 food items are grouped into four categories: cereal foods, sweets and snacks; dairy products, meats and fish; fruit; and vegetables. A separate set of questions covers intake of alcoholic beverages. The DQES takes approximately 30–45 minutes to complete. The DQES has been validated in mid-age Australian women [45] and a community sample of young Australian adults [46].

Sweetened drink intake (soft-drink, sports drinks, energy drinks, fruit drinks and cordial) will be measured using a separate three-item questionnaire as sweetened drink intake is not measured in the Cancer Council DQES. For each sweetened drink participants indicate how often, on average, they consumed the drink in the previous 12 months on a 10-point scale ranging from never to three or more times per day, and how much they usually consume on a 3-point scale comprised of one glass (250 ml), one can (375 ml) or one bottle (600 ml).

#### *Mediators of physical activity change*

The Physical Activity Maintenance Assessment (PAMA) will be used to measure participant motivation (10 items), self-efficacy (5 items) and confidence in maintaining physical activity (3 items). Responses will be measured on a 5-point Likert Scale ranging from ‘very unlike me’ to ‘very much like me’. Evidence shows that the PAMA has predictive validity with other accepted measures of physical activity (e.g. VO_2_ max and energy expenditure, as measured by the 7-Day Physical Activity Recall) and other health characteristics that are associated with regular physical activity (e.g., lower body mass index and reduced scores on the Beck Depression Inventory [47].

#### *Functional status and quality of life*

Functional status and quality of life will be measured using the Australian adaptation of the Short Form (36) Health Survey (SF-36) [48]. The SF-36 comprises 36 items representing eight domains of health and quality of life: vitality; physical functioning; bodily pain; general health perceptions; physical role functioning, emotional role functioning; social role functioning; and mental health. By combining these eight domains, two further subscales are derived: Physical Component Scale (PCS) and Mental Component Scale (MCS). Where necessary, scales are reverse-scored so that a higher score indicates better health. The SF-36 has been used in other studies of lifestyle interventions among cancer survivors and in self-management interventions in chronic disease [49,50]. Consistent with reliability analysis of the American version, all scales of the Australian version of the SF-36 demonstrate high internal consistency, with Cronbach’s alpha ranging from 0.81 (General Health) to 0.92 (Physical Functioning and Bodily Pain). The median of item-scale correlations within each scale ranged from 0.61 (Mental Health) to 0.82 (Role-Physical) [47].

#### *Demographics*

A brief questionnaire will be used to assess demographic characteristics including age, gender, marital status, ethnicity, level of education, cancer type, cancer stage at diagnosis and treatment type(s).

### Statistical considerations

#### *Sample size calculation*

A priori power calculations determined that a sample of 44 participants per group (n = 88) is needed to detect moderate effect sizes (r = 0.30-0.50) with 80% power and a significance level (alpha) of 0.05. Allowing for 20% dropout, 55 participants are required per group. Allowing for adjustment by baseline measure (r = 0.05) a total of 41 participants per group (n = 82) will be recruited.

#### *Analytic plan*

Data will be entered and analysed using the IBM SPSS version 21 (SPSS, Inc, Chacago, IL) statistical software package. The intent-to-treat principle will be applied and the significance level will be set at 0.05. Baseline comparisons of demographic outcomes will be performed using independent samples t-tests. The primary outcome (number of steps/day) will be reported as independent samples t-tests comparing change scores in the two randomisation groups. Change scores for the secondary outcomes (health measures, diet quality and quality of life) will be calculated for each variable and compared between treatment and control groups using parametric or non-parametric procedures as appropriate for the data distributions.

Random effects mixed modelling will be used with time (0, 12, and 24 weeks) and group allocation (Intervention, Control) as the fixed factors. A time by group interaction term will be used to formally test the aims of the study.

Time by region (rural versus metropolitan) repeated measures ANOVA will be used to examine differences in the primary and secondary outcomes between groups over time. Sociodemographic factors and diet will be controlled for in the analysis.

## Discussion

Cancer survivors commonly experience negative effects following cancer and its treatment(s), including fatigue, functional impairment, weight gain and cancer recurrence [2-6]. Engagement in regular physical activity may ameliorate or prevent such adverse effects and enhance quality of life [8-13]. This research involves the development and trial of an online resource to assist cancer survivors in both metropolitan and rural areas to become more physically active and to adopt a healthier diet. Currently, there are no existing step guidelines for cancer survivors. A value of 10,000 steps per day for healthy adults is often reported in the media as an appropriate goal. However, such a universal goal may not be achievable for those who have had cancer. The STRIDE study will contribute significantly to the literature as it will use RPE and daily affect to create individualised, incremental step goals relative to the individual’s baseline values. Thus the intervention will be tailored to the survivor’s current capacity for physical activity. The online delivery of the program will allow wide community reach and dissemination, which is particularly important among cancer survivors in rural communities who have reduced access to services and facilities compared to their urban counterparts. Specifically, the results from this study could form the basis of the development of a specific exercise prescription for this population.

## Abbreviations

STRIDE: Steps toward improving diet and exercise; RPE: Rating of perceived exertion.

## Competing interests

The authors declare that they have no competing interests.

## Authors’ contributions

All authors contributed to the protocol design and reviewed and edited the manuscript. LF, DZ and RS planned, coordinated and conducted the study. LF drafted the manuscript. All authors read and approved the final manuscript.

## Authors’ information

LF is a PhD student with the School of Health Sciences and a member of the Exercise for Health and Human Performance (EHHP) Group. DZ and RS are project managers, based in the EHHP group, within the School of Health Science at the University of South Australia. GP is an Associate Professor, based in the EHHP group, within the School of Health Science at the University of South Australia. JD is an Associate Professor, based in the EHHP group, within the School of Health Science at the University of South Australia.

## Pre-publication history

The pre-publication history for this paper can be accessed here:

http://www.biomedcentral.com/1471-2407/14/428/prepub
